# Free Flap Surgery for Elbow Soft Tissue Reconstruction Using the Brachial Artery as Recipient Vessel: Evaluation of MPETS Cases and Comparative Literature Review

**DOI:** 10.3390/medicina61020295

**Published:** 2025-02-08

**Authors:** Mitsutoshi Ota, Makoto Motomiya, Naoya Watanabe, Kazuya Kitaguchi, Norimasa Iwasaki

**Affiliations:** 1 Department of Orthopaedic Surgery, Obihiro Kosei Hospital Hand Center, Obihiro 080-0024, Japan; ota.mitsu.toshi.72@outlook.com (M.O.);; 2 Department of Orthopaedic Surgery, Faculty of Medicine and Graduate School of Medicine, Hokkaido University, Sapporo 060-0808, Japan; 3 Department of Radiological Technology, Obihiro Kosei Hospital, Obihiro 080-0024, Japan

**Keywords:** brachial artery, elbow, soft tissue defect, free flap, end-to-side anastomosis

## Abstract

*Background and Objectives:* The elbow joint, essential for daily activities, often requires soft tissue reconstruction following trauma, infection, or tumor excision. Free flap surgery using the brachial artery (BA) as the recipient vessel offers stable vascular support, but preserving distal blood flow is crucial. Due to vessel diameter differences, end-to-side (ETS) anastomosis is usually necessary, as flow-through anastomosis can be challenging. Although reports exist on soft tissue reconstruction using the BA as the recipient vessel, complications and outcomes related to using the sole main artery as the recipient remain unclear. We developed the microscopic parachute end-to-side (MPETS) technique, adapted from ETS, to more easily address vessel size discrepancies. This study evaluates the effectiveness and safety of MPETS in BA-based elbow reconstruction, alongside a review of outcomes in other cases. *Materials and Methods:* We retrospectively analyzed seven cases of elbow reconstruction from April 2018 to September 2023, focusing on patients with BA recipient vessels and a minimum 12-month follow-up. Variables included patient demographics, etiologies, flap types, and postoperative outcomes measured by Jupiter’s Criteria. Following PRISMA 2020 guidelines, a systematic literature review identified similar cases using the BA in free flap reconstruction for comparison. *Results:* In all our cases, flap survival was 100%, with no distal ischemia observed, and the average range of motion was 119°. Complications were limited, with one reoperation due to venous thrombosis. The MPETS technique minimized blood flow issues and accommodated the BA’s diameter. The literature review included 77 cases, confirming the BA’s viability and stability as a recipient vessel. *Conclusions:* Using the BA as a recipient vessel with MPETS demonstrates high effectiveness and safety in elbow soft tissue reconstruction. Our results support the BA’s suitability for complex reconstructions, with MPETS enhancing vessel compatibility and reducing complications.

## 1. Introduction

The elbow joint is a crucial structure in daily activities due to its complex anatomy and broad range of motion [1]. It requires coverage by soft tissues that provide durability and flexibility and contribute to the joint’s inherent bony stability [2,3]. Soft tissue defects around the elbow often arise from infection following total elbow arthroplasty, severe trauma, or tumor excision [4,5]. Timely and appropriate reconstruction is vital for controlling wound infection or osteomyelitis and preserving limb function [2,6]. The pedicled latissimus dorsi (LD) flap, known for its thin, flexible, and durable qualities, is a critical option for addressing such reconstruction [7]. However, challenges and limitations have been reported, including the potential loss of future functional and aesthetic reconstruction options [2], and the risk of ischemia at the distal end of the flap, with reports indicating partial necrosis in 38% of pedicled LD flaps extending beyond the olecranon [8]. Consequently, there is growing interest in using free flap surgery for reconstructing extensive soft tissue defects [5,9,10,11]. Various free flaps, such as the LD flap, rectus abdominis flap, anterolateral thigh (ALT) flap, and superficial circumflex iliac artery perforator (SCIP) flap, have been reported [9,12,13].

When performing free flap surgery, meticulous preoperative planning, including the selection of recipient vessels, is crucial to avoid severe complications [14]. In particular, severe trauma or infection-induced soft tissue defects in the limbs are more likely to cause refractory arterial spasms due to the “zone of injury,” compared to defects in the trunk [14,15,16,17]. Therefore, careful selection of recipient vessels is essential when performing free flap surgery around the elbow, with the brachial artery (BA) often being chosen as the recipient vessel [9,11,18,19]. When using the BA, a major artery, as the recipient vessel, it is critical to preserve distal blood flow. Due to the difference in vessel diameter, end-to-side (ETS) anastomosis is usually necessary, as flow-through anastomosis can be difficult to perform. Although there are reports on soft tissue reconstruction using the BA as the recipient vessel [20,21], complications and outcomes related to using the sole main artery as the recipient remain unclear.

We have previously developed the microscopic parachute end-to-side (MPETS) technique, adapted from the ETS vascular anastomosis method used in cardiovascular surgery, and reported its efficacy and safety [22,23,24,25]. This study aims to evaluate the effectiveness and safety of free flap surgery using the MPETS vascular anastomosis technique with the brachial artery as the recipient vessel in functional reconstruction around the elbow. Additionally, we aim to review the outcomes of previous reports that used the BA as the recipient vessel in elbow reconstruction.

## 2. Materials and Methods

### 2.1. Patients

**Figure 1:**
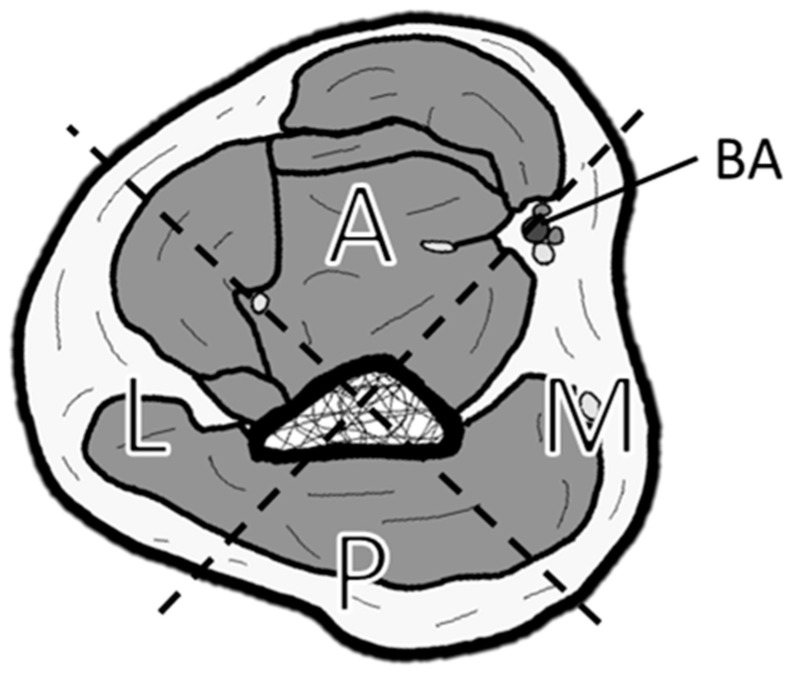
Classification of soft tissue defect locations around the elbow. The elbow is divided into four zones by drawing a line connecting the center of the humerus and the brachial artery (BA), and a perpendicular line to it: A (anterior), M (medial), P (posterior), and L (lateral).

This study was approved by our institutional ethics committee (approval number: 2024-039). From April 2018 to September 2023, our department performed free flap reconstructions in 123 patients, of which 84 cases involved the MPETS technique. We retrospectively analyzed all seven cases of elbow reconstruction performed during this period, focusing on cases that used the BA as the recipient vessel, with a mean follow-up period of 30 months (ranging from 15 to 66 months). We examined patients’ demographics, etiology, flap locations, flap types, the distance from the olecranon to the flap tip, flap size, outcomes based on Jupiter’s Criteria [26,27], and complications. The method for classifying soft tissue reconstruction locations is illustrated in Figure 1. Intraoperative vessel measurements were performed by saving images with a scale under the microscope, and these were measured postoperatively. Vessel size discrepancy was calculated by dividing the recipient vessel diameter by the flap pedicle diameter, and the expansion rate was determined by dividing the arteriotomy length by the flap pedicle diameter.

### 2.2. Surgical Techniques

**Figure 2:**
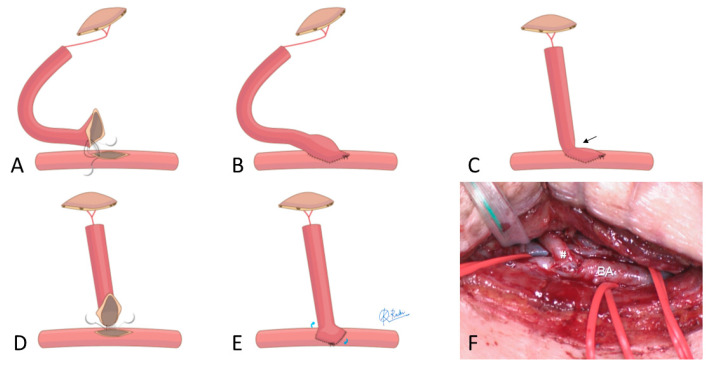
Tips for managing short vascular pedicles in the MPETS technique. (**A**,**B**): When the vascular pedicle is long, the vessel can be positioned at the anastomosis site without excessive tension. MPETS can be easily performed to align the flap vessels parallel to the recipient vessels. (**C**): When the vascular pedicle is short, kinking (arrow) at the anastomosis site can impair blood flow. (**D**,**E**): During vascular anastomosis, shifting the heel position horizontally along the slit, rather than positioning it at the tip, can help prevent stenosis at the anastomosis site. (**F**): The heel position (hashmark) was shifted horizontally when anastomosing the short vascular pedicle of the VFG to the BA (Case 1).

**Figure 3:**
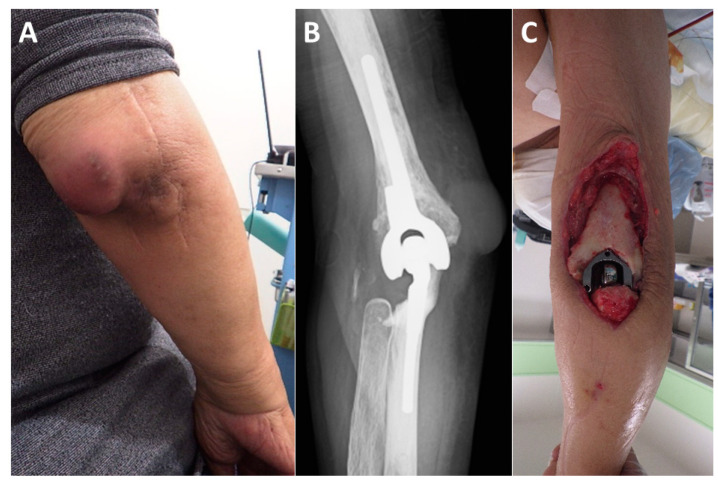

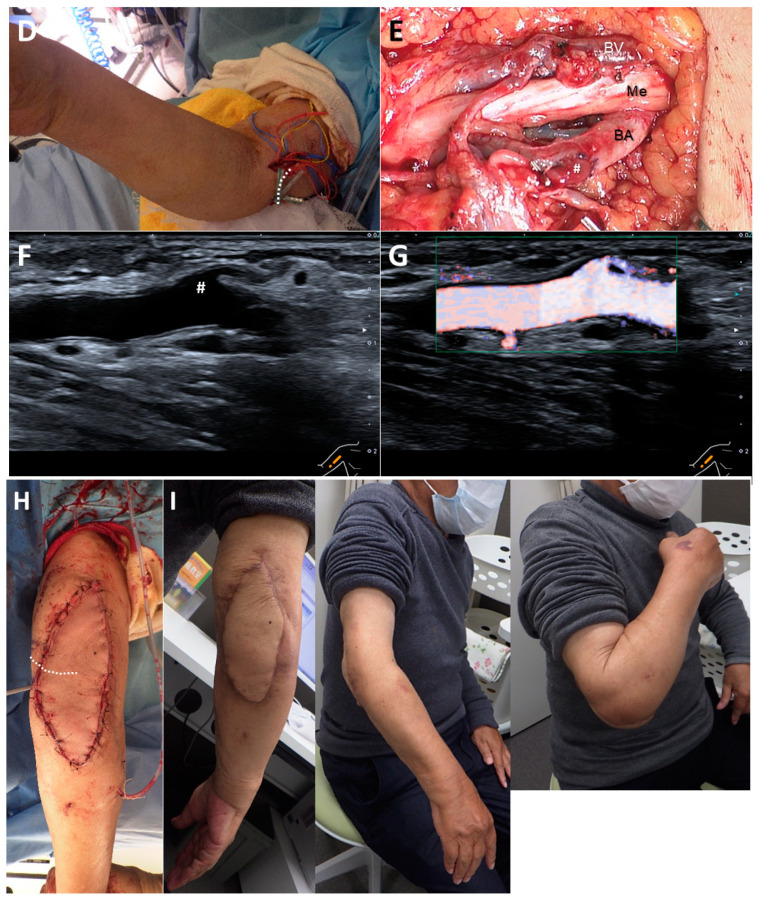
Representative case (Case 6). The patient is a 74-year-old male with rheumatoid arthritis. (**A**): Four years after total elbow arthroplasty, the patient developed an infection. (**B**): Plain radiographs show no evidence of implant loosening. (**C**): Despite multiple debridements and antibiotic therapy, which successfully controlled the infection, a large soft tissue defect developed on the posterior aspect of the elbow. (**D**): On the 14th day after the initial surgery, a free ALT flap was performed using the BA as the recipient vessel (flap size: 90 cm^2^). The flap pedicle vessels (dotted line) were guided through a spacious subcutaneous tunnel to the anterior aspect of the BA. (**E**): The flap pedicle vessels were aligned parallel to the BA and accompanying brachial vein (BV), ensuring a smooth transition towards the defect site (BA diameter: 5.5 mm, flap pedicle diameter: 1.5 mm). The anastomosed artery (hashmark) and vein (asterisk) can be seen bulging over the BA and accompanying vein. (Me; median nerve). (**F**): Ultrasound imaging at three weeks postoperatively shows a well-expanded anastomosis (hashmark). (**G**): Color Doppler imaging reveals no turbulence at the anastomosis site, with adequate blood flow in both the flap vessels and the distal recipient vessels. (**H**): The soft tissue defect is adequately covered by the ALT flap. (**I**): No postoperative complications were observed, and at the final follow-up one year after surgery, the patient had an elbow extension of −20° and flexion of 145°, with a “Good” outcome.

As part of preoperative planning, the location of the BA and accompanying veins closest to the reconstruction site was designated as the anastomosis site. To confirm vessel patency, color Doppler ultrasound was performed around the planned anastomosis site. To enable parallel alignment of the recipient vessels and flap pedicle, the flap pedicle was harvested to its maximum length (Figure 2A). With the patient in a supine position, the BA and accompanying veins were dissected between the biceps and brachialis muscles, preserving the median nerve (Figure 3D). All vascular anastomoses were performed using the MPETS technique (Figure 2) [22,23,24,25]. Briefly, the flap vessel was significantly widened, and a slit-shaped large window was created in the recipient vessel. Using the parachute technique, anastomosis was first performed at the heel, a high-risk site for blood leakage. Continuous suturing was then applied to the posterior and anterior walls to control bleeding from the window.

If the flap pedicle is sufficiently long, the MPETS can be easily performed to align the flap vessels parallel to the recipient vessels (Figure 2A,B and Figure 3E). In cases with a short pedicle, the heel position is shifted horizontally, and the anastomosis is performed midway along the slit-shaped window instead of at the tip, to prevent stenosis at the anastomosis site (Figure 2C–F) [23].

For the postoperative regimen, anticoagulation therapy was not administered in all cases. Instead, to improve peripheral circulation, PGE was administered at 40–80 µg/day for one week postoperatively at the surgeon’s discretion. Patients were prescribed bed rest for three days and were instructed to refrain from smoking for at least three weeks after surgery.

### 2.3. Ultrasonographic Vascular and Blood Flow Measurement

Approximately three weeks after surgery, a specialist in ultrasound imaging conducted blood flow assessments using Doppler ultrasound. The procedure followed the protocols established in earlier studies [23]. Patients were positioned supine on a bed and rested until their blood pressure stabilized. A high-resolution ultrasound machine (Aplio 1800, Canon Medical Systems Co., Ltd., Nasu, Japan) equipped with a linear probe operating at 12–18 MHz was used to measure the vessel diameter and blood flow at three specific locations: just proximal to the anastomosis site in the recipient artery, distal to the anastomosis site in the flap artery, and distal to the anastomosis site in the recipient artery. The transducer angle was maintained below 60 degrees during the measurements, with each measurement repeated three times and the average values recorded. Color Doppler mode was also employed to check for turbulence at the anastomosis site (Figure 3F,G). When mosaic flow was observed, it was considered suggestive of accelerated blood flow associated with stenosis. In cases where signal loss or retrograde flow was noted, it was interpreted as indicating blood flow interruption. For cases with accelerated blood flow, the peak systolic velocity was measured using the pulsed Doppler method, and cases in which the peak systolic velocity ratio across the stenotic region exceeded two times were defined as having abnormal velocity increase due to stenosis [28].

### 2.4. Literature Search Method

Following the PRISMA 2020 guidelines, a comprehensive literature search was conducted on 2 July 2024, using the PubMed, Web of Science, and EBSCOhost databases [29]. The search strategy included terms such as “free flap” AND “brachial artery” or “free flap” AND “elbow” to ensure the thorough retrieval of relevant case reports and publications available up to that date.

### 2.5. Study Selection Criteria

**Figure 4:**
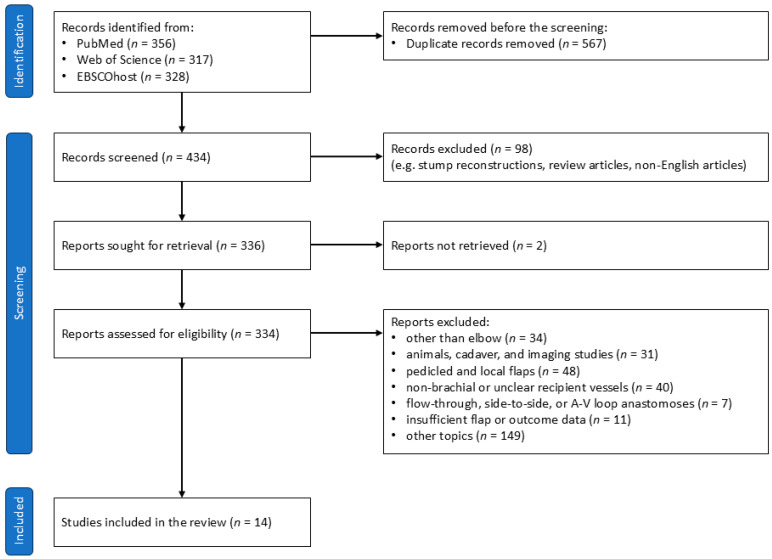
PRISMA 2020 flow diagram.

The study selection process, outlined in the PRISMA 2020 flow diagram, adhered to predefined inclusion and exclusion criteria (Figure 4). The inclusion criteria targeted case series involving free flaps around the elbow where the BA was used as the recipient vessel. The exclusion criteria eliminated studies on free tissue transfer for amputation stump reconstruction, review articles without specific case examples, non-English articles, non-retrievable articles, studies focused on regions other than the elbow, studies limited to animals, cadavers, or imaging, research on pedicled or local flaps, articles with unclear documentation of recipient vessels or those not using the BA, studies utilizing flow-through, side-to-side, or A-V loop vein grafts for recipient vessel anastomosis, papers without documented flap outcomes, and research unrelated to the primary theme.

### 2.6. Data Extraction and Synthesis

At the outset, two independent reviewers evaluated the titles and abstracts of the identified articles to determine their eligibility. Full texts were retrieved for studies deemed potentially suitable for inclusion, allowing for a more in-depth analysis. Data extraction focused on various study characteristics, including the author, publication year, and journal, as well as detailed patient information, such as demographics, etiology, flap type, complications related to the flap, anastomosis methods, and reconstruction sites. The analysis was conducted based solely on the data available. Case reports were qualitatively reviewed using the NIH assessment tool [30]. Any discrepancies between the reviewers were resolved through consensus or, if necessary, by consulting a third reviewer.

## 3. Results

### 3.1. Case Profiles and Flap Characteristics

All patient and flap background data are presented in Table 1. Among the seven cases of elbow reconstruction, four were due to trauma, two were due to infection, and one was due to post-traumatic joint contracture. The locations of the free flaps were as follows: two were confined to the anterior region (A), one to the lateral region (L), one to the posterior region (P), one spanned the anterior and lateral regions (A and L), one spanned the anterior and medial regions (A and M), and one extended from the anterior to the medial and posterior regions (A, M, and P). The flap types used were ALT in four patients, LD in two, and a vascularized fibula graft (VFG) in one. The recipient vessels in all cases were the BA and its accompanying veins. In Case 1, where a VFG was performed in the lateral region (L), the short pedicle required adjusting the heel position during the anastomosis to relieve tension on the vascular anastomosis site (Figure 2F).

The average size of the free flaps used was 151.5 cm^2^, and the average distance from the olecranon to the distal tip of the flap was 16.0 cm. Flap survival was 100%, with no adverse effects on distal extremity perfusion. The average time to wound healing was 15 days (ranging from 8 to 35 days). In one case (case 4), delayed wound healing was observed due to postoperative surgical site infection, requiring more than three weeks for complete healing. Since most cases involved reconstruction of soft tissue defects associated with crush injuries or infections, antibiotics were generally administered until wound exudate subsided. The average duration of antibiotic administration was 19 days postoperatively (ranging from 4 to 45 days). The average extension/flexion range of motion was −14°/133°, resulting in an arc of 119°, with outcomes classified as Excellent in two cases and Good in five cases based on Jupiter’s Criteria [26,27]. One case (case 4) required reoperation due to venous thrombosis that developed after inadvertent elbow flexion and extension during dressing changes on postoperative day four.

### 3.2. Vascular Measurements of Recipient and Flap Pedicles

Intraoperative measurements related to vascular anastomoses for all cases are presented in Table 2. The average diameter of the BA at the anastomosis site was 4.7 mm, while the average diameter of the flap artery was 2.3 mm, resulting in an average vessel size discrepancy of 2.3 times at the anastomosis site. The length of the arteriotomy averaged 5.8 mm, approximately 2.6 times the diameter of the flap artery. The average diameter of the brachial vein at the anastomosis site was 2.6 mm, while the average diameter of the flap vein was 2.1 mm, leading to an average vessel size discrepancy of 1.5 times at the anastomosis site. The length of the venotomy averaged 5.4 mm, approximately 3.0 times the diameter of the flap vein. Two venous anastomoses were performed in three cases.

Postoperative ultrasonographic data obtained three weeks after surgery are shown in Table 3. The average internal diameter of the BA, both proximal and distal to the anastomosis site, was 3.8 mm, while the average internal diameter of the flap artery was 2.1 mm, resulting in a vessel size discrepancy of 2.0 times at the anastomosis site. The average blood flow rate in the BA proximal to the anastomosis site was 142.1 mL/min, with the flap artery averaging 26.2 mL/min, and the BA distal to the anastomosis site maintaining a high flow rate, averaging 113.1 mL/min. No turbulence was observed at the anastomosis site in any of the cases.

### 3.3. Review Results

Our comprehensive literature search did not identify any comparative studies using the BA as the recipient vessel. Focusing on case reports and case series, we reviewed 15 papers, including our own report, which encompassed a total of 77 flap outcomes (Table 4). With data available for each parameter, the average age of the 68 patients was 38.5 years (ranging from 14 to 84 years), and the average postoperative follow-up period for the 41 patients was 65 months (ranging from 3 to 130 months). Regarding the etiology of soft tissue defects, trauma was the most common cause, accounting for 64.9% (50 out of 77 cases), followed by infection at 18.2% (14 out of 77 cases), and malignant tumors at 9.1% (7 out of 77 cases). Among the 44 cases with available data on flap placement, the anterior region (A) was the most common location, at 40.9% (18 out of 44 cases), followed by the posterior region (P) at 27.3% (12 out of 44 cases). The combination of the lateral and posterior regions (L and P) and the anterior and medial regions (A and M) each accounted for 6.8% (3 out of 44 cases), while circumferential coverage was noted in 4.5% (2 out of 44 cases).

The most frequently used flap type was classified as fasciocutaneous flaps, such as the ALT, accounting for 59.7% (46 out of 77 cases). Muscle flaps, including the LD and gracilis, were used in 37.7% (29 out of 77 cases). Osseous flaps, such as the VFG, were used in 2.6% (2 out of 77 cases). The average size of the flaps, based on 63 available cases, was 169 cm^2^. Reports regarding the vascular anastomosis technique were available in only 17% (13 out of 77 cases), with ETS using a long-axis slit vesselotomy on the recipient vessel in all cases. Postoperative anticoagulation therapy was rarely administered, with intravenous dextran infusion and oral aspirin administration given in 2.6% (2 out of 77 cases). Complications included complete flap necrosis in 1.3% (1 out of 77 cases) and partial flap necrosis in 3.9% (3 out of 77 cases). Arterial occlusion occurred in 3.9% (3 out of 77 cases) and venous thrombosis in 5.2% (4 out of 77 cases), with reoperation performed for these vascular occlusions. Postoperative surgical site infection was observed in 5.2% (4 out of 77 cases) and delayed wound healing in 15.6% (12 out of 77 cases). The average postoperative range of motion in the elbow, based on 41 available cases, was 101.2°. No cases of distal ischemia were reported following ETS anastomosis.

## 4. Discussion

In this study, we investigated the use of the BA as a recipient vessel for free flap surgery in functional reconstruction around the elbow. We were the first to highlight the BA’s characteristics as a recipient vessel, including its large diameter, high blood flow, and the significant size discrepancy between the BA and the flap pedicle vessels. Haddock et al. [38] outlined the ideal characteristics of a recipient vessel: adequate diameter, stable anatomy, consistent positioning, ease of exposure, patient positioning, and minimal sacrifice. The BA meets these criteria, making it a reliable recipient vessel as evidenced by several reports of free flap surgeries utilizing the BA [9,11,18,19,20,21,31,32,33,34,35,36,37,39]. However, there are few detailed reports on specific anastomosis techniques or considerations when using this high-flow, large-caliber artery.

Understanding the characteristics of the BA and its suitability for various reconstructive sites and functions is crucial in recognizing the BA as a valuable recipient vessel in elbow reconstruction. The elbow’s intricate three-dimensional anatomy often requires reconstruction across multiple regions. The BA, positioned from the medial to the anterior aspect of the elbow, necessitates careful planning to prevent twisting of the flap pedicle during reconstruction. While vascular placement is straightforward in anterior and medial reconstructions due to the planar relationship between the flap and the BA, lateral and posterior reconstructions require three-dimensional positioning of the pedicle from the graft site to the anastomosis site. Surgical planning that considers the positional relationship between the flap and the BA is essential, especially when reconstructing in the posterior (P) or lateral (L) regions, as we demonstrated.

Only two previous reports, by Chui et al. [9] and Gerakopoulos et al. [34], have specifically addressed vascular anastomosis techniques, both of which used the long-axis slit technique described by Tan et al. [40]. Our MPETS technique, similar to Tan’s method, involves creating a long-axis slit in the recipient vessel. This approach allows for an oblique, rather than perpendicular, alignment of the anastomosis, facilitating a smooth transition in vessel diameter, particularly when considering the BA’s location on the medial side of the elbow. Koteswara et al. [11] reported arterial failure caused by significant atherosclerosis in the recipient artery during vascular anastomosis with vessel diameter discrepancies. Although the details of the anastomosis technique were not provided, it is known that the long-axis slit technique is suitable for arteries with severe atherosclerosis [41], suggesting that this method could have been a viable approach in addressing the issue. This technique is considered a beneficial method for free flap surgeries using the BA as a recipient vessel in elbow reconstructions, even when vascular conditions are suboptimal.

The MPETS technique offers several advantages for BA-to-flap ETS anastomoses. Generally, heel anastomosis in ETS procedures is considered technically challenging [42,43]. However, the MPETS method allows for precise anastomosis by carefully suturing the heel portion while the vessels remain separated and then approximating them using the parachute technique. In our study, the size discrepancy between the BA and the flap pedicle vessels was an average of 2.3 times. However, by enlarging the flap vessel’s end and matching it with the slit made in the artery, the anastomosis diameter was expanded to an average of 5.8 mm (approximately 2.6 times the original flap vessel diameter). The MPETS technique effectively accommodates the size discrepancy between the large BA and the smaller flap pedicle vessels. As shown in Figure 2, when the flap pedicle is short, there is an increased risk of stenosis at the anastomosis site. To address this in one of our cases (Case 1), we adjusted the heel position by shifting it horizontally and suturing it along the middle section of the slit, rather than at the end. This technique proved to be effective in preventing stenosis [23]. Therefore, the MPETS technique demonstrates flexibility and adaptability, even in more complex anastomosis scenarios.

Our comprehensive literature review revealed that free flap surgeries using the BA as a recipient vessel around the elbow have been employed for a variety of conditions, including severe trauma, serious infections, and tumor excision. In these cases, microvascular vessels are often unsuitable as recipient vessels due to the high likelihood of being encased in scar tissue, making them difficult to expose, or due to vasospasm caused by the “zone of injury” [14,15,16,17]. Given the thin skin and large range of motion around the elbow (especially the olecranon), coverage of extensive soft tissue defects, as observed in the cases we reviewed (average flap size 169 cm^2^), is essential. The BA’s course in the superficial medial upper arm allows for flexible adjustment of the anastomosis site according to the target coverage area. Furthermore, by using flaps that can accommodate long pedicles, such as ALT or LD, effective coverage was achieved in areas farther from the BA, like the posterior (P) or lateral regions (L), in 39% of cases (17 out of 44 cases).

Only Koteswara et al. [11] and our study assessed the extent to which the coverage extended distally from the olecranon, with an average of 14.2 cm and 16.0 cm, respectively. This is beyond the typical reach of pedicled latissimus dorsi flaps, which generally extend only 8–8.4 cm distal to the olecranon [44,45]. A range of motion of 100° is generally considered necessary for daily activities [46], and many reports have demonstrated good postoperative ranges of motion (average 101.2°) without elbow extension contracture [9,11,18,19,21,33,34,35,36,37,39].

Flap loss is a recognized complication of free flap surgery. The overall flap loss rate for free flaps in lower extremity soft tissue reconstruction is reported to be 6.0% [47]. In contrast, the flap loss rate for free flaps in upper extremity reconstruction is reported to be 4.3–6.0% [48,49]. In our investigation of free flaps using the BA as the recipient vessel, the total flap loss rate was 1.3%, with no noticeable increase in the incidence of partial flap loss or delayed wound healing. Additionally, no cases of serious complications such as distal ischemia were reported. These findings suggest that the BA is a safe and effective recipient vessel for functional reconstruction around the elbow.

Regarding the venous route, the BA is accompanied by a large, stable deep vein (average diameter 2.6 mm), which was used as a venous recipient in all seven of our cases. Securing an appropriate venous route can be challenging in patients with a history of multiple surgeries [50], and when there is a discrepancy in vessel size between the recipient vein and the flap vein, performing end-to-end anastomosis can further increase the risk of flap failure [51]. End-to-side anastomosis of the deep vein accompanying the artery is advantageous as it allows for easy preoperative planning, minimizes the surgical field, and is associated with lower complication rates and greater safety [25,52]. In three of the reviewed reports, the artery was anastomosed to the BA, and the vein was anastomosed to the accompanying vein using the ETS method [20,35,36]. Anastomosing both the main artery and its accompanying vein using the MPETS technique allows for straightforward preoperative planning, reduces intraoperative complications, and ensures consistent anastomosis outcomes regardless of vessel size discrepancies.

In one of our cases, a reoperation was required due to venous thrombosis caused by inadvertent elbow flexion during postoperative dressing changes. While functional reconstruction of the elbow with a free flap offers a favorable postoperative range of motion, it is crucial to ensure the following: (1) an adequate length of the pedicle to allow for sufficient slack, (2) avoidance of vascular anastomosis near the flexion center of the cubital fossa, and (3) verification of the safe range of joint motion relative to the anastomosed vessels during surgery.

This study has several limitations that should be considered. First, the sample size of seven cases is relatively small, which limits the generalizability of the findings. Conducting the study at a single center may introduce selection bias, making it difficult to apply the results to other clinical settings. Additionally, the retrospective design may have limitations in data accuracy, and the absence of a control group makes it challenging to directly compare the use of the BA as a recipient vessel with other options. Furthermore, while the MPETS technique showed promise in addressing the challenges of BA anastomosis, the lack of comparison with other anastomosis techniques limits the comprehensive evaluation of its relative advantages.

Despite these limitations, this study contributes significantly to the field of functional reconstruction. It is one of the first to rigorously evaluate the use of the BA as a recipient vessel in free flap surgery around the elbow, providing valuable insights into its efficacy and safety. The detailed assessment of the MPETS technique adds to the existing body of knowledge, particularly in cases involving large-caliber vessels like the BA. The study’s focus on both intraoperative and postoperative parameters offers a comprehensive evaluation of the surgical process and its effectiveness.

Future research should involve larger, multicenter studies to validate these findings and further evaluate the efficacy of the MPETS technique. Comparative studies of different anastomosis techniques and recipient vessels will also be essential for optimizing surgical approaches in elbow reconstruction.

## 5. Conclusions

In this study, we examined seven cases of free flap surgery for functional reconstruction around the elbow, utilizing the BA as the recipient vessel. We thoroughly evaluated the placement, size, vascular anastomosis techniques, and postoperative outcomes of the free flaps. Our findings, along with a review of existing literature, reaffirm the validity and safety of using the BA as a recipient vessel. This study demonstrates that elbow reconstruction can be effectively achieved by using the BA in combination with flaps that have long pedicles or muscle flaps. Mastery of end-to-side anastomosis is crucial, and the MPETS technique has proven to be a reliable and safe method for vascular anastomosis. The use of the BA as a recipient vessel in free flap surgery represents a valuable and important option for functional reconstruction around the elbow.

## Figures and Tables

**Table 1:** Case profiles and flap characteristics.

No.	Age	Sex	BMI	Underlying Condition	Smoking	Etiology	Location of Flap *	Flap Type	Flap Size (cm^2^)	Extent of Flap Beyond Olecranon (cm)	Complication	Elbow E/F (°)	Jupiter’s Criteria
1	57	M	24.3	DM, CKD, COPD	Current	Infection (chronic humeral osteomyelitis)	L	VFG	20	N/A	None	−10/120	Good
2	84	F	23.9	Psy	Never	Trauma (Gustilo IIIB)	A	ALT	60	15	None	−25/135	Good
3	54	M	21.0	None	Current	Trauma (Gustilo IIIB)	A/L	LD	221	17	None	−20/125	Good
4	45	M	23.9	None	Past	Trauma (Gustilo IIIC)	A/M	LD	230	22	Flap venous thrombosis Delayed wound healing Surgical site infection	0/130	Excellent
5	54	M	24.9	HT	Past	Other (contracture after trauma)	A	ALT	90	N/A	None	10/135	Excellent
6	74	M	23.8	HT, RA	Past	Infection (after TEA)	P	ALT	90	5	None	−20/145	Good
7	22	F	16.6	None	Never	Trauma (Gustilo IIIB)	A/M/P	ALT	350	21	None	−30/140	Good
Ave	55.7		22.6						151.5	16.0		−14/133	

BMI, body mass index; E/F, extension/flexion; DM, diabetes mellitus; CKD, chronic kidney disease; COPD, chronic obstructive pulmonary disease; VFG, vascularized fibula graft; Psy, psychiatry; ALT, anterolateral thigh; LD, latissimus dorsi; HT, hypertension; RA, rheumatoid arthritis; TEA, total elbow arthroplasty; Ave, average; *, In “Location of Flap”, the abbreviations indicate the orientation of the flap relative to the elbow joint: A (anterior), M (medial), P (posterior), and L (lateral).

**Table 2:** Intraoperative measurements of recipient vessels and flap pedicles.

	Artery					Vein				
No.	Recipient Diameter (mm)	Flap Diameter (mm)	Length of Arteriotomy (mm)	Vessel Size Discrepancy	Expansion Rate of Flap Pedicle	Recipient Diameter (mm)	Flap Diameter (mm)	Length of Venotomy (mm)	Vessel Size Discrepancy	Expansion Rate of Flap Pedicle
1	4.0	3.0	6.0	1.3	2.0	4.0	3.0	6.0	1.3	2.0
						3.4	2.8	8.0	1.2	2.9
2	4.5	2.5	7.0	1.8	2.8	2.0	1.0	4.0	2.0	4.0
						3.5	1.5	6.0	2.3	4.0
3	3.6	2.2	6.0	1.6	2.7	2.0	3.0	4.0	0.7	1.3
4	5.5	2.8	6.0	2.0	2.1	1.8	2.5	6.0	0.7	2.4
5	5.6	1.4	3.5	4.0	2.5	1.5	1.0	3.5	1.5	3.5
						2.2	1.2	4.2	1.8	3.5
6	5.5	1.5	5.5	3.7	3.7	3.0	1.2	5.0	2.5	4.2
7	4.0	2.5	6.5	1.6	2.6	2.3	3.3	7.0	0.7	2.1
Ave	4.7	2.3	5.8	2.3	2.6	2.6	2.1	5.4	1.5	3.0

Ave, average.

**Table 3:** Postoperative measurements of recipient arteries and flap arteries.

No.	Proximal Recipient Vessel Diameter (mm)	Proximal Recipient Flow Volume (mL/min)	Flap Pedicle Diameter (mm)	Flap Pedicle Flow Volume (mL/min)	Vessel Size Discrepancy	Distal Recipient Vessel Diameter (mm)	Distal Recipient Flow Volume (mL/min)
1	3.8	115.7	3.0	28.3	1.3	3.8	99.0
2	3.9	120.2	2.2	3.2	1.8	3.8	103.6
3	3.6	131.0	2.0	24.0	1.8	3.3	93.3
4	4.3	246.0	1.9	75.3	2.3	4.3	175.0
5	3.8	97.0	1.0	3.7	3.8	3.8	83.0
6	3.7	117.7	2.0	2.3	1.9	4.2	99.7
7	3.2	167.0	2.5	46.7	1.3	3.2	138.3
Ave	3.8	142.1	2.1	26.2	2.0	3.8	113.1

Ave, average.

**Table 4:** Summary of previous reports on free flaps using the brachial artery as the recipient vessel for soft tissue reconstruction around the elbow.

Author (Year)	Number of Flaps	Age	Etiology	Location of Flap ^#^	FLAP TYPE	Size (cm^2^)	Complication	Postoperative ROM (°)	Quality Assessment Score
Chui et al. (2012) [9]	5	39.4 (22–61)	Trauma (*n* = 3)	P (*n* = 3)	ALT (*n* = 5)	199 (36–450)	Surgical site infection (*n* = 2)	102 (45–140)	7/9
			Infection (*n* = 2)	M/P (*n* = 1)			Heterotopic ossification (*n* = 1)		
Koteswara et al. (2019) [11]	9	NA (14–71)	Trauma (*n* = 9)	A (*n* = 2)	ALT (*n* = 6)	255 (120–540)	Complete flap loss (*n* = 1)	NA	5/9
					ALT + RF (*n* = 2)		Flap arterial failure (*n* = 1)	>90 (*n* = 2)	
					FL (*n* = 1)		Partial flap loss (*n* = 1)	50–90 (*n* = 3)	
							Recipient-site hematoma (*n* = 1)	<50 (*n* = 1)	
McGraw et al. (2024) [18]	21	43.0 (23–68)	Trauma (*n* = 12)	P (*n* = 2)	ALT (*n* = 15)	107.5 (50–250)	Delayed wound healing (*n* = 10)	NA	7/9
			Infection (*n* = 3)		LD (*n* = 3)		Seroma (*n* = 2)	>120 (*n* = 8)	
			Malignancy (*n* = 1)		TRAM (*n* = 1)		Surgical site infection (*n* = 1)	60–120 (*n* = 6)	
			Other (*n* = 5)		Scapular (*n* = 1)		Donor-site hematoma (*n* = 1)	<60 (*n* = 3)	
					Parascapular (*n* = 1)		Flap venous thrombosis (*n* = 1)		
Bezirgan et al. (2022) [19]	8	29.5 (18–43)	Trauma (*n* = 4)	P (*n* = 5)	ALT (*n* = 8)	125 (80–352)	Partial flap loss (*n* = 1)	133 (100–145)	7/9
			Malignancy (*n* = 4)	A/M/P/L (*n* = 1)					
Kagaya et al. (2020) [20]	1	74	Malignancy (*n* = 1)	A/M (*n* = 2)	LD (*n* = 1)	162	-	NA	5/9
Kilmartin et al. (2018) [21]	1	64	Trauma (*n* = 1)	A/L/P (*n* = 1)	VFG (*n* = 1)	300	-	105	6/9
Hamdi et al. (2004) [31]	5	34.2 (15–47)	Infection (*n* = 5)	P (*n* = 1)	TAP (*n* = 2)	203 (126–252)	Flap venous thrombosis (*n* = 2)	NA	6/9
				L/P (*n* = 1)	LD + TAP (*n* = 2)		Delayed wound healing (*n* = 1)		
					DIEP (*n* = 1)				
Ng et al. (2016) [32]	1	68	Malignancy (*n* = 1)	A/M/P/L (*n* = 1)	LD (*n* = 1)	240	-	0 (arthrodesis)	7/9
di Summa et al. (2019) [33]	2	30.5 (18–43)	Trauma (*n* = 2)	N/A	ALT + FL (*n* = 1)	188 (171–204)	-	123 (120–125)	7/9
					ALT + VL + FL (*n* = 1)				
Gerakopoulos et al. (2021) [34]	1	54	Trauma (*n* = 1)	L (*n* = 1)	ALT + FL (*n* = 1)	NA	-	100	6/9
Coulet et al. (2011) [35]	12	25.6 (23–37)	Trauma (*n* = 12)	A (*n* = 12)	Gracilis (*n* = 12)	N/A	Flap arterial thrombosis (*n* = 2)	70 (0–120)	7/9
							Partial flap loss (*n* = 1)		
Wechselberger et al. (2009) [36]	1	22	Trauma (*n* = 1)	A (*n* = 1)	RF (*n* = 1)	N/A	-	110	6/9
Wade et al. (2020) [37]	1	32	Trauma (*n* = 1)	A (*n* = 1)	Gracilis (*n* = 1)	72	-	125	5/9
Our study	7	55.7 (22–84)	Trauma (*n* = 4)	A *(n* = 2)	ALT (*n* = 4)	152 (20–350)	Flap venous thrombosis (*n* = 1)	119 (105–145)	7/9
			Infection (*n* = 2)	L (*n* = 1)	LD (*n* = 2)		Delayed wound healing (*n* = 1)		
			Other (*n* = 1)	P (*n* = 1)	VFG (*n* = 1)		Surgical site infection (*n* = 1)		
				A/L (*n* = 1)					
				A/M (*n* = 1)					
				A/M/P (*n* = 1)					

ALT, anterolateral thigh; DIEP, deep inferior epigastric perforator; FL, fascia lata; LD, latissimus dorsi; RF, rectus femoris; TAP, thoracodorsal perforator; TRAM, transverse rectus abdominis myocutaneous; VFG, vascularized fibula graft; VL, vastus lateralis; #, In “Location of Flap”, the abbreviations indicate the orientation of the flap relative to the elbow joint: A (anterior), M (medial), P (posterior), and L (lateral).

## Data Availability

The data presented in this study are available on request from the corresponding author due to ethical restrictions.

## Abbreviations

The following abbreviations are used in this manuscript:

BA	brachial artery
ETS	end-to-side
MPETS	microscopic parachute end-to-side
LD	latissimus dorsi
ALT	anterolateral thigh
SCIP	superficial circumflex iliac artery perforator
VFG	vascularized fibula graft
